# Ginger Constituents and its Effects on Gastrointestinal malignancies: A Review of clinical trials

**DOI:** 10.1093/eurpub/ckac131.235

**Published:** 2022-10-25

**Authors:** L Aregawi, Z Csiki, M Shokralahi, H Teame

**Affiliations:** 1 Institute of Nutrition, University of Debrecen, Debrecen, Hungary; 2 Public Health, Adigrat University, Adigrat, Ethiopia

## Abstract

The gastrointestinal tract (GI tract) is the tract of the digestive system that contains all the major organs of the digestive system. Disorder in any part of the GI tract results in various disease forms such as malignancies. The occurrence of GI cancer is very high in developed countries including Europe. A wide variety of natural products containing anticancer properties showed significant effects. Ginger, the rhizome of Zingiber officinale, is among one of the natural herbal remedies widely used for its spice and medicinal properties. It is fair sources of vitamins like, β-carotene, vitamin C and minerals. Recently ginger has shown significant attention in clinical studies due to its anti-cancer effects that possesses promising potential for inhibiting the proliferation of multiple cancer cells. The aim of this review is to provide a summarized report of clinical trials on ginger constituents and its effects on gastrointestinal malignancies. A systematic search was conducted by two independent authors on the databases of Scopus, Clinical Trials, PubMed and Science with the search term of the key words. This review article supported that ginger is an important plant with several constituents and gastrointestinal medicinal effects. Ginger constituents suppress the growth and induce apoptosis of variety of cancer types including colon, gastric, pancreatic and other GI cancers. It is mainly the 6-gingerol and 6-shogaol, of the major compounds in ginger rhizomes, among hundreds of molecules. It is reported that antioxidant and anti-inflammatory properties of ginger support its preventive role against the gastrointestinal malignancies.

**Key messages:**

• This review article provided evidences supporting the effects of ginger on gastrointestinal malignancies and demonstrates the importance of future studies.

• Therefore, more extensive and well-controlled clinical trial studies of ginger are required to demonstrate its effect on gastrointestinal malignancies.

